# Characterization of Thermally Treated Gas-Atomized Al 5056 Powder

**DOI:** 10.3390/ma13184051

**Published:** 2020-09-12

**Authors:** Kyle Tsaknopoulos, Caitlin Walde, Derek Tsaknopoulos, Victor Champagne, Danielle Cote

**Affiliations:** 1 Material Science and Engineering Program, Department of Mechanical Engineering, Worcester Polytechnic Institute, 100 Institute Road, Worcester, MA 01609, USA; cewalde@wpi.edu (C.W.); dgtsaknopoulos@wpi.edu (D.T.); dlcote2@wpi.edu (D.C.); 2 US Army Research Laboratory, Aberdeen Proving Ground, MD 21005-5069, USA; victor.k.champagne.civ@mail.mil

**Keywords:** powder, aluminum, cold spray, Al 5056, atomization, rapid solidification

## Abstract

Aluminum 5056 is a work-hardenable alloy known for its corrosion resistance with new applications in additive manufacturing. A good understanding of the secondary phases in Al 5056 powders is important for understanding the properties of the final parts. In this study, the effects of different thermal treatments on the microstructure of Al 5056 powder were studied. Thermodynamic models were used to guide the interpretation of the microstructure as a function of thermal treatment, providing insight into the stability of different possible phases present in the alloy. Through the use of transmission electron microscopy (TEM) and energy-dispersive X-ray spectroscopy (EDS), combined with thermodynamic modeling, a greater understanding of the internal microstructure of Al 5056 powder has been achieved in both the as-atomized and thermally treated conditions. Evidence of natural aging within these powders was observed, which speaks to the shelf-life of these powders and the importance of proper treatment and storage to maintain consistent results.

## 1. Introduction

Al 5xxx series alloys were developed in the 1930s to meet a need for higher strength sheet materials with good formability, weldability, and corrosion resistance [1]. Magnesium shortages during World War II decreased the production of Al-Mg alloys until the 1950s, when new alloys were created with easier formability due to the increase of manufacturing capabilities. These new Al 5xxx series alloys could be used in many forms, including castings, extrusions, plates, sheets, and wires. These alloys are the only non-heat-treatable aluminum alloys to be used as plates or casts [1]. Specifically, Al 5056 is used for automotive applications, weld wire, welded storage vessels, rivets for magnesium, and fasteners [2,3]. Due to its high corrosion resistance, this alloy is often used on parts that are exposed to marine environments, such as boat hulls and gangplanks [2].

In powder form, the Al 5056 alloy has been used substantially in the solid-state metal additive manufacturing process of cold spray. In this process, powder is carried by an inert gas through a nozzle at supersonic velocities towards a substrate, where the particles plastically deform and adhere to create bonded layers [4]. Since this alloy is work-hardenable, work hardening effects of the cold spray process can be leveraged, making Al 5065 a logical choice for a feedstock material [5]. There have been two main applications of Al 5056 powder in cold spray. The first is a composite coating of Al 5056 with SiC. This composite mixture leads to reinforcement of the aluminum matrix to improve hardness and wear resistance, and properties can be controlled through the ratio of SiC to Al 5056 in the powder [6,7,8]. The addition of SiC particles was also shown to reduce the porosity in the coating. Since cold spray can only be used on ductile materials, the use of Al 5056 powder allows for ceramic particles to be used in the cold spray process [9]. The second application is the use of Al 5056 for cold spray repair of magnesium rotorcraft components [9,10,11]. The alloy was chosen to help make improvements to the corrosion resistance of the magnesium alloy [10]. The magnesium rotorcraft parts generally failed due to galvanic corrosion. Since Al 5056 has a high magnesium content, it was chosen to help reduce this phenomenon. Al 5056 powder has also been used as a cold-sprayed transition material between a magnesium alloy and Al 6061 substrates using fraction stir welding [12].

Properties and performance of the cold-sprayed coating are a function of the secondary phases in the feedstock powder. These secondary phases are dependent on exact alloy composition and are formed by phase transformations occurring during powder solidification, formation, powder processing, cold spray processing, and in-service conditions. In aluminum alloys, the addition of magnesium yields higher strength values, due to solid solution strengthening, that are directly proportional to the magnesium content in ranges up to 6 wt% [3]. An increased magnesium content increases the strength without excessively decreasing the ductility [2]. Titanium is added as a grain refiner, while manganese and chromium can correct for the corroding effect of iron, which is an impurity in aluminum alloys [13]. 

In wrought Al 5056, since the aluminum matrix is supersaturated with magnesium, there is a high driving force for the precipitation of the β-Al_3_Mg_2_ phase [1]. Additionally, due to the low solubility of Mg_2_Si in the aluminum matrix for this alloy, Mg_2_Si can be seen as a major phase. The presence of chromium in this alloy leads to the formation of sub-microscopic intermetallic particles in the form of Al_12_Mg_2_Cr. The addition of manganese leads to the formation of sub-microscopic Al_6_(Mn,Fe), as well as larger versions of that intermetallic. The presence of these intermetallics does not adversely affect its corrosion resistance; however, coarsened manganese and chromium particles can reduce ductility [2,13]. Al-Fe coarse intermetallic particles, including Al_12_(Fe,Mn)_3_Si and Al_3_Fe, reduce ductility as well as creep and fatigue resistance. When the silicon solute is bound in Mg_2_Si in this alloy, manganese precipitates as an additional phase, favorably as Al_6_(Mn,Fe) over other aluminum–iron phases [1,13]. 

While in service, thermal variations can affect the microstructure. While the aluminum matrix is supersaturated with magnesium, it is mostly stable at room temperature, but precipitation of β-Al_3_Mg_2_ can be accelerated by deformation and elevated temperatures, where it then precipitates on grain boundaries and shear bands. If β-Al_3_Mg_2_ forms at high temperatures (above 260 °C), stable β-Al_3_Mg_2_ will form, but β’ will form first at room temperature. β’ is very stable at low temperatures and does not readily evolve to equilibrium phases, even after long aging times. Precipitation of β-Al_3_Mg_2_ leads to softening; when precipitated at grain boundaries, it decreases corrosion resistance as it acts as an anodic phase to the matrix [1]. Additionally, the presence of β-Al_3_Mg_2_ at grain boundaries leads to intergranular cracking and stress corrosion [2]. Furthermore, the Al_18_Mg_3_Cr_2_ phase and the Al_6_(Mn,Fe) phase can precipitate during ingot preheating, and therefore special attention should be given to these phases during thermal changes [1,2].

Since Al 5056 is not an age-hardenable alloy, it is typically not thermally treated in the conventional way. However, literature has shown that applying a solution treatment with subsequent natural aging, while not increasing the strength of the alloy, can double the percent elongation of the alloy [13]. Research on Al 5056 powder for cold spray has shown that heat treatment at 400 °C for 6 h has been used to degas the powder to reduce sintering during processing [11]. This process also led to a more homogenous distribution of magnesium in the matrix. This study will investigate the effect of thermal treatment on the internal microstructure of Al 5056 powder.

## 2. Materials and Methods 

Commercially available gas-atomized Al 5056 powder (Valimet, Stockton, CA, USA) was studied; the composition was in compliance with ASTM B316 (Table 1) [14]. Size and shape analysis of the powder was conducted using the Microtrac Flow Sync Synchronous Laser Diffraction (Microtrac MRB, Montgomeryville, PA, USA) and Dynamic Image Particle Analyzer (Microtrac MRB, Montgomeryville, PA, USA).

**Figure 1:**
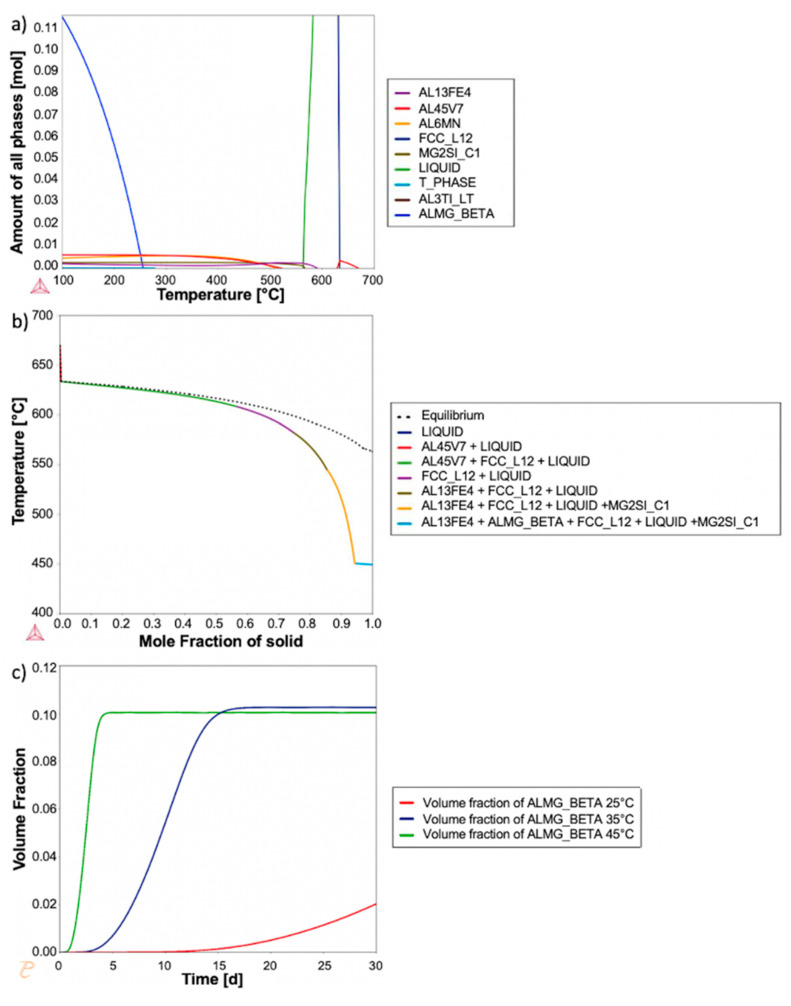
Thermodynamic models produced using Thermo-Calc. (**a**) Equilibrium diagram and (**b**) Scheil (non-equilibrium) solidification diagram. (**c**) Volume fraction as a function of time for β-Al_3_Mg_2_ precipitate.

Thermodynamic and kinetic modeling software can aid in understanding the structure and processing of metallic alloy systems. A commercially available software, Thermo-Calc (Thermo-Calc 2019a, Solona, Sweden), was utilized to create equilibrium and Scheil solidification diagrams (Figure 1) using the TCAl5 Aluminum database and the measured chemical composition of the Al 5056 powder shown in Table 1. All measured elements were included in the model calculations. Other elements were not taken into account as none were detected in excess of 0.01%. The Scheil Calculator with default settings was used to create the Scheil solidification diagram. The Equilibrium Calculator with default settings and a system size of 1 mole was used to create the equilibrium diagram. These diagrams provided insight into the internal microstructure of the powder by predicting secondary phases present in various conditions.

While Al 5056 is typically not considered a heat-treatable alloy, solution treatment can be applied to homogenize the microstructure. The equilibrium property diagram (Figure 1a) was consulted when choosing a treatment temperature. A temperature of 530 °C was chosen; it is below the melting temperature and above the dissolution temperature of Mg_2_Si for this specific composition. A treatment time of 1 h was chosen as it has been shown that the treatment times for gas-atomized powders are much shorter than typical thermal treatment times [16,17,18,19]. A differential scanning calorimeter (DSC) with a Liquid Nitrogen Pump Accessory (LN2P) cooler (Discovery DSC, TA Instruments, New Castke. DE, USA) was used to thermally process the powder, due to the high level of control of the heating and cooling rates, at a heating rate of 50 °C/min and a cooling rate of 120 °C/min with a nitrogen purge gas. 

It has been seen that SEM can be insufficient to show secondary phases of a few micron diameter; therefore, transmission electron microscopy (TEM) is preferred [16,17,18,19,20] to observe the submicron phases. Samples were created for TEM imaging using a gallium focused ion beam (FIB) (FEI Helios 660 Nanolab and FEI Scios Dual Beam FIBs) in a method similar to that employed by Tsaknopoulos et al. [17]. Parallel-sided lamellas of the powder particles of varying dimensions were produced. For energy-dispersive X-ray spectroscopy (EDS) elemental quantification, samples below 100 nm in thickness were created to limit interaction of the matrix and other phases with the phase under consideration. 

TEM and STEM micrographs were collected using a probe-corrected TEM with ChemiSTEM technology (Titan Themis 300 S/TEM, Thermo Fisher Scientific, Waltham, MA, USA), and EDS micrographs were collected using a Super-X EDS system (Super X EDS, Thermo Fisher Scientific, Waltham. MA, USA). All analyses were performed at 300 kV.

## 3. Results and Discussion

**Figure 2:**
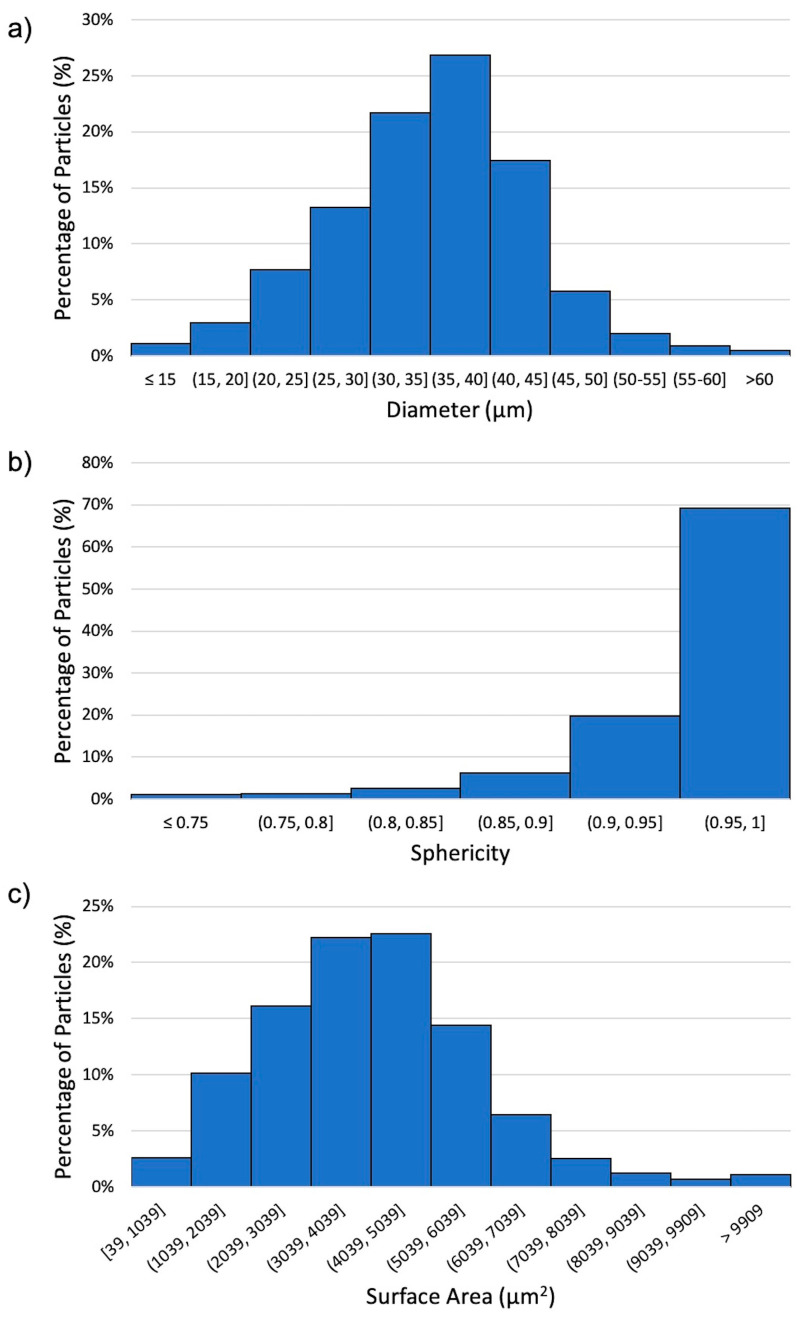
(**a**) Histogram of diameter of Aluminum 5056 powder particles; (**b**) histogram of sphericity of Aluminum 5056 powder particles; (**c**) histogram of surface area of Aluminum 5056 powder particles.

The diameter, sphericity, and surface area of Al 5056 powder particles were measured with a Microtrac Flow Sync. Figure 2a shows a histogram of the diameter measurements for the Al 5056 powder. The distribution showed a d_10_ of 22.3 μm, a d_50_ of 30.75 μm, and a d_90_ of 41.85 μm. The Aluminum 5056 powder particle diameter was found to have a mean of 35.1 μm and a standard deviation of 8.4 μm, with a minimum of 3.5 μm and a maximum of 185 μm. Figure 2b shows a histogram of the sphericity measurements of the powder particles. The sphericity was found to have a mean of 0.95 with a standard deviation of 0.05, a minimum of 0.33, and a maximum of 0.99. Figure 2c shows a histogram of the surface area of the particles. The mean was found to be 4090 μm, with a standard deviation of 1940 μm, a minimum of 38 μm, and a maximum of 107,000 μm. Most commonly, the cold spray process uses powders in the 5–100 μm range, with special cases using both smaller and larger particles [4]. Some work has been done on the effects of particle size on critical impact velocity for cold spray, but further studies are needed to understand both the ideal shape and morphology of powder for cold spray and their effects on consolidated cold spray properties [10,21]. 

**Figure 3:**
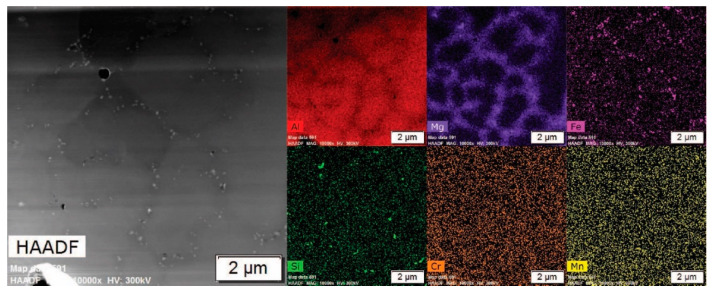
Low-magnification transition electron microscopy (TEM) micrographs of the as-atomized condition: HAADF and energy-dispersive X-ray spectroscopy (EDS) maps.

**Figure 4:**
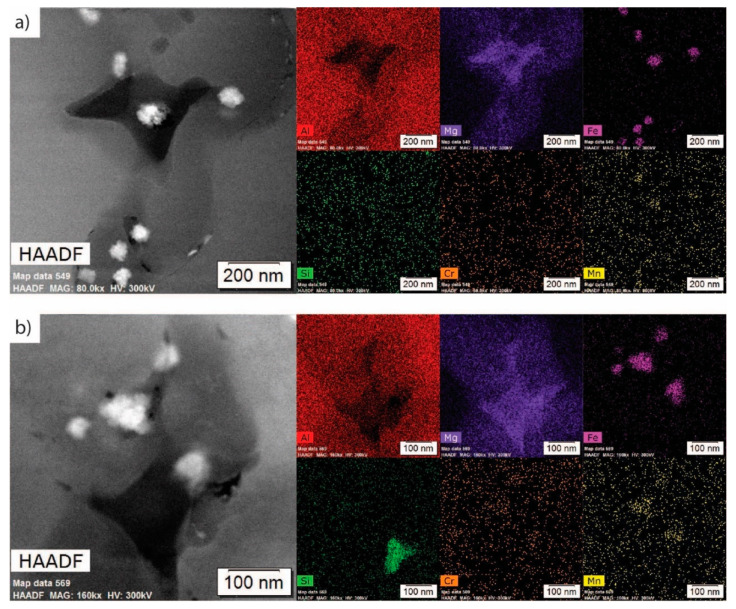
High-magnification TEM micrographs of the as-atomized condition showing (**a**) a grain boundary with an Fe intermetallic (light contrasting) and an Al-Mg phase (dark contrasting), as well as Mg segregation; (**b**) a grain corner featuring the same Fe intermetallic (light contrasting), a Mg-Si phase (dark contrasting), and two morphologies of the Al-Mg phase.

Figure 3 displays a low-magnification high-angle annular dark-field (HAADF) and EDS maps representative of the as-atomized Al 5056 internal microstructure; regions of magnesium solute segregation, dispersed iron intermetallic phases at the grain boundaries, and some Mg-Si phases at the grain boundaries are seen. Figure 4a shows a STEM HAADF micrograph and corresponding STEM EDS maps at a high magnification of the grain boundary. This demonstrates the presence of flower-like disks of an iron intermetallic, as well as a dark contrasting Al-Mg phase. These are dispersed in the magnesium solute segregation in this alloy, which was caused by its very high magnesium content. The difference between the magnesium segregation and the Al-Mg phase is evident in the magnesium EDS maps. Figure 4b shows a similar micrograph with the presence of a Mg-Si phase at the grain corner. A combination of thermodynamic modeling and EDS elemental quantification can be used to propose the identity of the different phases. The Scheil solidification diagram in Figure 1b demonstrates the presence of Al_13_Fe_4_, β-Al_3_Mg_2_, and Mg_2_Si for alloy composition. It has been shown that Scheil solidification predictions can be valid for powders due to their rapidly solidifying nature [16,17,18,19]. Comparing the predicted phases and EDS results, the Fe intermetallic is likely Al_13_Fe_4_, the Al-Mg phase is likely β-Al_3_Mg_2_, and the Mg-Si phase is likely Mg_2_Si. This is further supported with elemental quantification, where the Fe intermetallic is approximately 79 atm% Al (with the remainder being Fe), which is close to the 76 atm% Al of Al_13_Fe_4_. The Al-Mg phase is approximately in the 60 atm% Al and 40 atm% Mg proportions of β-Al_3_Mg_2_, and the Mg-Si phase is approximately 68 atm% Mg and 32 atm% Si, corresponding to the stoichiometry of Mg_2_Si. The magnesium solute segregation seen at the grain boundaries can be homogenized using thermal treatment to improve the properties of the powder [11].

**Figure 5:**
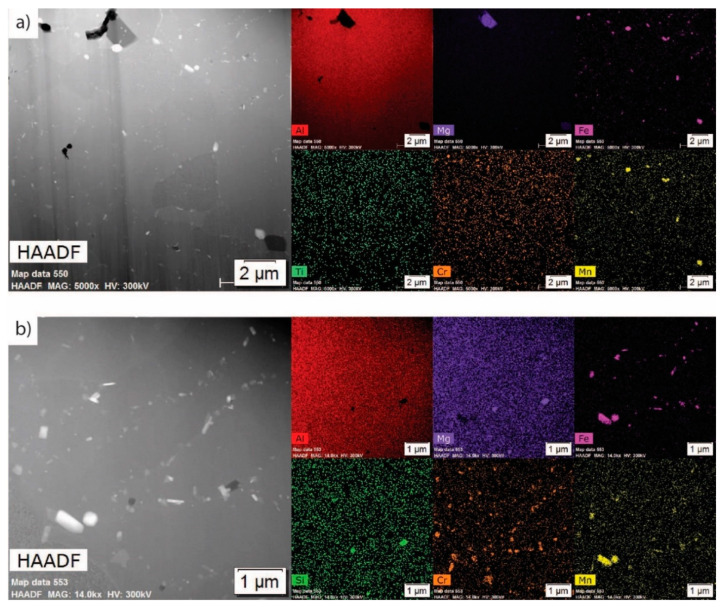
TEM micrographs and corresponding EDS maps of the thermally treated condition for (**a**) low magnification and (**b**) high magnification.

Based on the previous understanding of the phases in the as-atomized powder, further deductions can be made about the evolution of the powder microstructure with thermal treatment. A thermal treatment of 530 °C for 1 h was evaluated in this study. Figure 5 shows STEM HAADF micrographs and corresponding EDS maps of the microstructure of the thermally treated condition. Note the presence of some residual coarsened Mg-Si phase at the grain corners, while most of it has dissolved. Additionally, magnesium solute segregation at the grain boundaries has equilibrated throughout the matrix, and the Al-Mg phase has dissolved. Note the large and small bright contrasting phases at the grain boundaries in the HAADF image. The majority of these bright contrasting phases are likely coarsened Al_13_Fe_4_, with the largest phases present at the grain corners. The EDS maps and elemental quantification also denote an increase in Mn content of the Al-Fe phase, which is consistent with the stoichiometry for the Al_13_Fe_4_ phase found in Thermo-Calc, where Mn can substitute for Fe in this phase.

Based on the equilibrium diagram in Figure 1a, Mg_2_Si is stable in very small amounts at the treatment temperature of 530 °C; this is consistent with what was seen in the micrographs. Figure 1a demonstrates that β-Al_3_Mg_2_ will dissolve at 250 °C, which corresponds with the dissolution seen in the micrographs. Additionally, Figure 1a indicates that Al_13_Fe_4_ is more stable at the treatment temperature, thus leading to phase coarsening at this temperature, which is consistent with the coarsening seen in the micrographs. It has been shown that the homogenization of these powders leads to softening, which then leads to improved deformation and bonding in cold spray deposits [11]. Additionally, research has shown that coarse Al-Fe precipitates have low-strength bonds with the matrix, causing the material to be more brittle, and can act as crack initiation sites, both of which negatively affect the mechanical properties [22,23]. Given this, it is important to heat treat the powder to homogenize the magnesium solute segregation to improve ductility without excessive coarsening of the iron intermetallic. Thermodynamic models can be used further to optimize the thermal treatment parameters to yield the desired internal microstructure for the best cold spray properties.

Due to the supersaturated solid solution in Al 5056, natural aging can occur at room temperature. In the as-atomized condition of this sample, natural aging was seen in the form of nanosized Al-Mg particles of about 10–20 nm; these are likely the same β-Al_3_Mg_2_, as it is extremely stable at room temperature (as seen in Figure 1a). These are generally seen nucleating on the boundaries of other phases as well as along grain boundaries, identified as the small black contrasting dots in Figure 4a,b. Unlike the larger Al-Mg phases that formed during initial atomization, this Al-Mg phase has likely formed at room temperature over a longer period of time. Like the larger Al-Mg phase seen in the figures, the small Al-Mg phases also dissolve during heat treatment.

As shown in Figure 5a, the Al-Mg phase dissolved during heat treatment. After quenching, the sample was left at room temperature two days prior to being analyzed in the TEM. During this time, natural aging also occurred. During thermal treatment, as the phases in the as-atomized condition dissolved, the excess solute was homogenized into the matrix, further increasing the driving force for precipitation at room temperature due to the supersaturated matrix. Based on Figure 1a, it is predicted that the naturally aged phases could be β-Al_3_Mg_2_, Al_45_Cr_7_, Al_6_Mn, Mg_2_Si, or T-phase at room temperature. Figure 5b shows a higher magnification STEM HAADF and a corresponding EDS map for the micrograph in Figure 5a and demonstrates some of the naturally aged phases. Note the presence of many small (50 nm) chromium-rich phases at the grain boundaries. Figure 1 suggests that these would be Al_45_Cr_7_, but EDS quantification indicated the presence of magnesium in these phases; this potentially suggests a different Al-Cr intermetallic, possibly Al_12_Mg_2_Cr. The presence of what is potentially β-Al_3_Mg_2_ was again seen growing on the coarsened Al-Fe intermetallics, while small amounts of what is potentially T-phase were seen growing along grain boundaries. No apparent Al_6_Mn was seen in the TEM lamella, but it might be seen for other powder particles of this alloy composition, as suggested by Figure 1, because Al_6_Mn is stable at room temperature in small amounts. It has been shown that for Al 5xxx series alloys, natural aging of these phases at the grain boundaries increases stress corrosion cracking [24,25,26]. This is relevant when considering the shelf-life of powder, particularly after thermal treatment before use in consolidation processes, such as cold spray. Thermodynamic and kinetic modeling can be used to simulate and understand the natural aging behavior of this alloy. For example, Figure 1c shows the volume fraction of the β-Al_3_Mg_2_ phase as a function of time at various temperatures. At room temperature (25 °C), this phase slowly grows over the course of a month. With an increasing temperature, the growth rapidly accelerates until 45 °C, where growth is much faster, plateauing to maximum precipitation in a matter of days. These variations can affect the mechanical properties and repeatability of the additively manufactured parts. This model simulates conditions in a laboratory (highly controlled), or in a large warehouse (large temperature fluctuations), enabling a better understanding of the environmental impacts on the natural aging of β-Al_3_Mg_2_ in Al 5056.

In order to fully understand the phase transformations between the as-atomized and thermally treated conditions, hot-stage TEM will be performed in future work to evaluate the formation or dissolution of these phases in situ. Special consideration will be given to the natural aging seen in this alloy.

## 4. Conclusions

Understanding the microstructural changes in powders is important for predicting and understanding the mechanical and corrosion properties of the final additively manufactured parts in which they are used. Thermodynamic modeling in combination with TEM and EDS has allowed for a deeper understanding of microstructural evolution within Al 5056 powder for solid-state additive manufacturing applications in both the as-atomized and thermally treated conditions. The TEM and EDS unveiled the presence of magnesium segregation, an Al-Mg phase, a Mg-Si phase, and an Al-Fe phase in the as-atomized powder condition. The combination of thermodynamic modeling and EDS quantifications has suggested that these phases are likely β-Al_3_Mg_2_, Mg_2_Si, and Al_13_Fe_4_. Even though Al 5056 is not considered a heat-treatable alloy, literature has shown that a solution treatment and subsequent aging can increase the elongation percentage in these alloys. Given the high deformation experienced by powder particles during the cold spray process, a high elongation percentage is desirable. Additionally, a degassing heat treatment of the powders prior to use in the additive manufacturing process has been shown to improve the properties of the final consolidated part. Given this, a solution treatment of 530 °C for one hour was applied to the powders. After thermal treatment, the magnesium segregation and Al-Mg dissolved, and the Al-Fe and Mg-Si phases coarsened. This combination of concurrent dissolution and growth of phases demonstrates the need for thermal treatment optimization in order to homogenize segregation and detrimental Al-Mg phases while avoiding extreme coarsening of Al-Fe and Mg-Si phases. Additionally, after 2–14 days, there was evidence of natural aging, with the presence of small Al-Mg and Al-Cr phases. This speaks to the shelf-life of these powders after thermal treatment and the importance of proper treatment and storage to maintain consistent results.

## Figures and Tables

**Table 1:** Composition of Al 5056 powder as determined via direct current plasma emission spectroscopy [15].

Element	Wt%
Chromium	0.15
Copper	0.013
Iron	0.11
Magnesium	5.38
Manganese	0.16
Silicon	0.050
Zinc	0.005
Total others	<0.01
Aluminum	Remainder
